# Molecular and Biochemical Characterization of Recombinant Guinea Pig Tumor Necrosis Factor-Alpha

**DOI:** 10.1155/2015/619480

**Published:** 2015-04-27

**Authors:** Vijaya R. Dirisala, Amminikutty Jeevan, Lan H. Ly, David N. McMurray

**Affiliations:** ^1^Department of Microbial Pathogenesis and Immunology, College of Medicine, Texas A&M Health Science Center, College Station, TX 77843, USA; ^2^Department of Biotechnology, Vignan's University, Guntur 522213, India

## Abstract

Tumor necrosis factor alpha (TNF-*α*) is a cytokine which plays opposing roles in the context of infectious disease pathogenesis. TNF-*α* is essential for the development of a protective immune response to some pathogens, for example, *Mycobacterium tuberculosis*, by synergizing with other cytokines. However, exorbitant or uncontrolled TNF-*α* activity may also drive pathology and disease symptoms in many infectious diseases. In order to elucidate the beneficial and detrimental roles of TNF-*α* in tuberculosis (TB) and other diseases for which the guinea pig is the small animal model of choice, recombinant guinea pig (rgp)TNF-*α* has been produced using prokaryotic expression systems. However, it is unknown whether posttranslational modifications which cannot be made in the prokaryotic expression systems may be important for rgpTNF-*α* structure and function. Therefore, we carried out a comparative study by expressing rgpTNF-*α* in prokaryotic and eukaryotic expression systems and analyzed the eukaryotic-expressed rgpTNF-*α* for the presence of posttranslational modifications by subjecting it to NanoLC-MS/MS. We conclude that the eukaryotic-expressed rgpTNF-*α* lacks posttranslational modifications, and we found no significant difference in terms of the biological activity between prokaryotic- and eukaryotic-expressed rgpTNF-*α*. Taken together, results from our study show that a prokaryotic expression system can be used for generating large amounts of rgpTNF-*α* without concern for the biological integrity.

## 1. Introduction

Tumor necrosis factor alpha (TNF-*α*) plays important and contradictory roles in the pathogenesis of many infectious diseases, including tuberculosis (TB) [1, 2]. TNF-*α* synergizes with other cytokines in contributing to a protective immune response in the host in TB by promoting the formation and maintenance of granulomatous lesions which are considered to be an essential part of the host's attempts to control both the local accumulation and dissemination of the pathogen [3, 4]. Defective granuloma formation was observed in TNF-deficient mice infected with virulent* Mycobacterium tuberculosis* [5]. Humans treated with TNF-blocking drugs are at high risk of developing reactivation TB, reinforcing the critical role of TNF-*α* in the maintenance of host resistance [6]. On the other hand, uncontrolled TNF-*α* contributes to disease symptoms (e.g., fever and weight loss), tissue destruction, and organ pathology in TB and other chronic diseases [7]. Understanding these apparently contradictory functions of TNF-*α* will require the necessary reagents to study the molecule in both* in vitro* and* in vivo* studies in the small experimental animals of choice. Animal models such as mice, guinea pigs, rabbits, and monkeys are widely used in TB research [8]. The guinea pig model of pulmonary TB mimics human TB in many important ways, including the formation of typical, human-like granulomas, and other characteristic features which makes it a gold standard for evaluating novel vaccine candidates during preclinical trials [9]. Our laboratory has cloned and expressed several guinea pig cytokine and chemokine genes such as interleukin-8 (IL-8/CXCL-8) [10], regulated upon activation, normal T-cell expressed and secreted (RANTES/CCL5) [11], interferon-gamma (IFN-*γ*) [12], interleukin-4 (IL-4) [13], interleukin-10 (IL-10) [14], interleukin-1beta (IL-1beta) [15], monocyte chemoattractant protein-1 (MCP-1) [16], and interleukin-17 [17]. We have previously reported the generation of rgpTNF-*α* using a prokaryotic expression system [18] and have used this reagent to study the contributions of TNF-*α* to the response of both phagocytic cells and whole animals to infection with virulent* M. tuberculosis* [19].

The rgpTNF-*α* can be produced using either prokaryotic or eukaryotic expression systems. The advantages of prokaryotic expression systems are that a large amount of recombinant protein can be produced without the complication of maintaining large volumes of eukaryotic cell culture and purifying the protein from a complex matrix composed of other eukaryotic proteins [19]. On the other hand, eukaryotic expression systems have the advantage that the proteins produced may undergo posttranslational modifications which are required for their structural and biological integrity [20]. Posttranslational modifications were observed in cytokine and chemokine genes of humans and other species [21, 22].

All of our previous work with rgpTNF-*α* has been carried out with protein produced by* E. coli* [17, 23]. However, rgpTNF-*α* has not been generated using a eukaryotic expression system and the impact of posttranslational modifications on the structure and activity of this molecule remains to be determined. Therefore, in this study, we generated rgpTNF-*α* using an efficient eukaryotic expression system, analyzed the resulting protein for the presence of posttranslational modifications, and compared the biological activities of prokaryotic- and eukaryotic-expressed rgpTNF-*α*.

## 2. Materials and Methods

### 2.1. Prokaryotic Expression of Guinea Pig TNF-Alpha

The cloning of guinea pig TNF-*α* was accomplished by using the Concanavalin A-stimulated guinea pig splenocytes as described previously [24]. The construct containing coding sequence of guinea pig TNF-*α* was a generous gift from Dr. Teizo Yoshimura, National Cancer Institute, USA. The mature peptide region of guinea pig TNF-*α* (accession number-AF119622) was subcloned into the BamHI and HindIII site of pQE-30 vector (Qiagen, Chatsworth, CA) and transformed with M15 competent cells as described previously by our group [17]. Fresh transformants were obtained by streaking M15 bacterial culture containing subcloned guinea pig TNF-*α* in pQE-30 vector on Luria-Bertini (LB) agar plates containing 100 *μ*g/mL ampicillin (Sigma, St. Louis, MO) and 100 *μ*g/mL kanamycin (Sigma).

One of the transformants was inoculated into 5 milliliter (mL) of Difco-Luria-Bertini (LB) broth containing appropriate antibiotics and grown overnight at 37°C. 5 mL of the overnight culture was added to 100 mL of identical culture medium in a 250 mL flask the following day and was cultured on a shaker (220 rpm) at 37°C. When the OD_600_ of the culture reached 0.6, protein expression was induced by adding isopropyl-*β*-d-thiogalactoside (IPTG; Sigma) to a final concentration of 1.0 mM, followed by incubation for 5 hours at 37°C.

The cells were harvested by centrifugation and the pellet was resuspended in 5 mL lysis buffer following the manufacturer's instructions [25]. The sample was sonicated and centrifuged to obtain the cleared lysate that contains rgpTNF-*α*.

The cleared lysate was purified by Immobilized Metal Affinity Chromatography (IMAC) employing the Ni-NTA matrix (Qiagen) as described previously for other soluble recombinant guinea pig proteins [15]. All the purification steps were performed at 4°C. The nickel-charged Ni-NTA agarose resin was added to the cleared lysate in a polypropylene column (Qiagen) in a ratio of 1 : 4 and placed on an orbital shaker for 1 hour. After equilibrating the column with 5 mL lysis buffer, the column was subjected to washing twice with 10 mL of wash buffer, followed by final elution of the His-tagged protein by the addition of the elution buffer (5 mL). A small fraction of the eluted samples were run on Novex 10–20% Tricine gel (Invitrogen, Carlsbad, CA) and the gel was stained with Coomassie brilliant blue. The eluted fractions containing TNF-*α* were pooled and concentrated using Amicon centrifugal filter devices (Millipore) and the concentrated protein content was estimated using the Bradford assay kit (Bio-Rad).

### 2.2. Eukaryotic Expression of Guinea Pig TNF-*α*

The eukaryotic expression vector pCEP-Pu [26] used in this study does not contain the His tag. In order to add the His tag, the mature peptide region of TNF-*α* cloned into BamHI and HindIII restriction sites of the pQE-30 vector were amplified with primer sequences (Invitrogen) designed to contain NheI/XhoI recognition sequences so that, upon amplification from the 5′ end, the product contained the NheI flanking sequence-His tag-mature peptide region of the guinea pig TNF-*α*-Xho I flanking sequence. The forward and reverse primers containing NheI and XhoI recognition sequences used for amplification were 5′-TA**G CTA GC** *G CAT CAC CAT CAC CAT CAC GGA*-3′ and 5′-TA**C TCG AG** *C AAG CTT CTA GTT TGT TAA TTT*-3′. The italicized parts of the primer sequences are complementary to the nucleotide sequences of the guinea pig TNF-*α* cDNA whereas the 5′ overhangs (bolded and underlined) are flanking restriction sites designed to facilitate cloning. The amplified products were digested with the NheI/XhoI enzymes (New England Biolabs), and gel eluted prior to ligation with the pCEP-Pu vector containing the same restriction sites. The ligated product was used to chemically transform XL1-Blue competent cells (Agilent Technologies, Santa Clara, CA) according to the manufacturer's instructions and the presence of the inserts in the transformants was analyzed by restriction analysis with NheI and XhoI and subjected to Sanger sequencing.

### 2.3. Transfection of pCEP-Pu Vector Containing the TNF-*α* Gene

Human embryonic kidney (HEK) 293-EBNA cells (Invitrogen) grown in Dulbecco's Modified Eagle Medium (Invitrogen) according to our previously published procedure [14] were grown to three-fourth confluency and transfected for 24 h with Lipofectamine 2000 (Invitrogen) using different concentrations of pCEP-Pu plasmid DNA containing the gpTNF-*α* cDNA. The transfection medium was then replaced with CD-293 medium without serum containing puromycin (0.05 *μ*g/mL) and the cells were incubated at 37°C for 48 h before collecting the supernatant fluid. Purification of cell lysates containing the putative rgpTNF-*α* was performed in a similar manner to that described above for prokaryotic expressed rgpTNF-*α*.

### 2.4. Validation of Eukaryotic Expressed rgpTNF-*α* by Western Blot Analysis

The generation of polyclonal rabbit antiserum against prokaryotic-expressed rgpTNF-*α* has been described earlier [18]. In brief, the rgpTNF-*α* was mixed with the adjuvant TiterMax Gold (CytRx Corp, Norcross, GA) and injected subcutaneously into New Zealand white rabbits in four injections that were spaced at 3-week intervals. The animals were exsanguinated 5 weeks following the last booster and sera were collected by centrifugation of blood at 1500 rcf for 20 minutes. The serum was aliquoted and stored at −80°C until it was used in the Western blot assay.

Approximately 200 ng of eukaryotic-expressed rgpTNF-*α* protein was run on a 10–20% tricine gel (Invitrogen) and blotted onto a nitrocellulose membrane using the semidry Minitrans blot electrophoretic transfer cell apparatus (Bio-Rad). The identity of the eukaryotic-expressed rgpTNF-*α* was determined by its reaction to the polyclonal anti-rgpTNF-*α* antiserum by Western blot analysis using the WesternBreeze chromogenic kit (Invitrogen) following the manufacturer's instructions with anti-rabbit (IgG) antibody as the secondary antibody (Invitrogen).

### 2.5. LC-MS/MS Analysis of Eukaryotic-Expressed rgpTNF-*α* for Posttranslational Modifications

The eukaryotic-expressed rgpTNF-*α* (approximately 500 nanograms) was run on a SDS-Tricine gel and the band corresponding to rgpTNF-*α* was excised from the gel and subjected to nano LC-MS/MS.* In silico* digestion analysis was performed to judge the protease best suited for obtaining peptides in the correct mass range prior to subjecting it to LC-MS/MS [27]. Protein digests were separated by capillary rp-HPLC prior to in-line analysis of their masses and fragmentation patterns. Final data generated were analyzed using Scaffold software (http://www.proteomesoftware.com/Scaffold/Scaffold_viewer.htm).

### 2.6. Biological Activity of Prokaryotic- and Eukaryotic-Expressed rgpTNF-*α*

The biological activity of prokaryotic- and eukaryotic-expressed rgpTNF-*α* was analyzed by measuring their cytotoxicity on L929 fibroblasts as previously described [28]. In brief, the L929 cell suspension in RPMI 1640 without phenol red and supplemented with 2 *μ*M L-glutamine, 100 Units of penicillin/mL, 100 *μ*g of streptomycin/mL, and 2% FBS, at a concentration of 4 × 10^5^ cells/mL, were seeded in 100 *μ*L aliquots onto 96-well plates and incubated at 37°C overnight in a 5% CO_2_ incubator. The following day, 50 *μ*L of serially diluted rgpTNF-*α* samples and 50 *μ*L of an 8-*μ*g/mL actinomycin D solution (final concentration, 2 *μ*g/mL) were added to each well and incubated for an additional 20 h. Tetrazolium reagent (WST-1; Dojindo, Kumamoto, Japan), and 1-methoxymethyl phenazinium methyl sulfate (Dojindo) were dissolved at 6 and 0.4 mM, respectively, in phosphate-buffered saline (PBS). These were mixed at a ratio of 1 : 1, and 20 *μ*L was added to each well. The cells were incubated for 2 hours in a 5% CO_2_ incubator at 37°C to allow for the color reaction to develop and the reaction was terminated by adding 25 *μ*L of 1 N sulphuric acid (H_2_SO_4_). The optical density at both 450 nm (OD_450_) and 630 nm (OD_630_) in each well was measured with a microplate reader for the test and reference wavelengths. The net change (net OD_450_ − OD_630_) for each well was calculated as follows: net OD_450_ − OD_630_ = [(OD_450_ − OD_630_ of test well) − (OD_450_ − OD_630_ of TNF-*α*-treated control)]. Data obtained was analyzed with a standard curve generated using recombinant human TNF-*α* (R&D Systems Inc., Minneapolis, Minn.).

## 3. Results

### 3.1. Confirmation of Prokaryotic Expressed rgpTNF-*α*

Upon streaking on LB plate supplemented with antibiotics, bacterial transformants containing the cloned guinea pig TNF-*α* gene were obtained. Upon induction with IPTG, those transformants, generated of a protein that was visible as a 17 kDa band on a tricine gel. The rgpTNF-*α* protein was purified under native conditions using Ni-NTA columns, as the protein was obtained in the soluble form and was confirmed by its specific binding to antiserum obtained from rabbits immunized with prokaryotic expressed rgpTNF-*α* protein as described previously by our group [18].

### 3.2. Confirmation of Eukaryotic-Expressed rgpTNF-*α*

**Figure 1:**
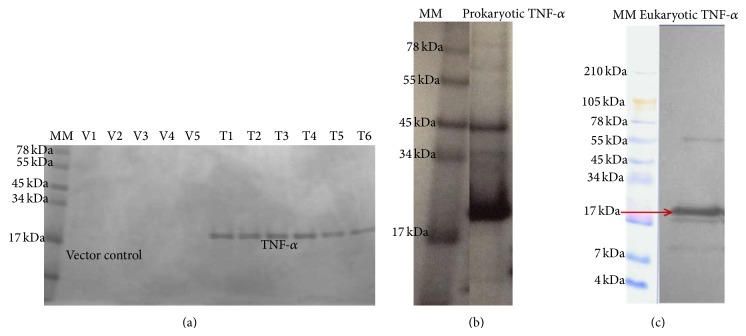
(a) Coomassie blue-stained SDS-PAGE analysis of eukaryotic protein elutions from cells transfected with 18 *μ*g of pCEP-Pu plasmid DNA without any gene (vector control) or pCEP-Pu plasmid DNA (18 *μ*g) containing TNF-*α* gene. The arrow indicates a ~19 kDa band which was recognized as rgpTNF-*α*. Absence of band in the vector control is also shown. MM: molecular marker; V: vector control elutions, and T: TNF-alpha elutions. (b) Coomassie blue-stained SDS-PAGE analysis of prokaryotic expressed rgp-TNF-*α*. rgpTNF-*α* was expressed using prokaryotic expression system (pQE-15) and analyzed on SDS-PAGE gel. MM: molecular marker. (c) Identification of eukaryotic expressed rgpTNF-*α* by polyclonal antiserum (1 : 2000 dilution) from immunized rabbits. Approximately 200 nanograms of rgpTNF-*α* was run on 10–20% tricine gel and analyzed by western blot analysis for checking its specificity. The ladder was stained with Coomassie Brilliant Blue.

Transformation of pCEP-Pu with the mature peptide region of the gpTNF-*α* gene residing in the pQE-30 vector resulted in the generation of putative transformants. All five clones that were randomly selected for restriction digestion analysis confirmed the presence of the insert. Plasmid DNA sequencing of these clones revealed that the TNF-*α* sequence was cloned in-frame with the His tag and thrombin cleavage site of pQE-30 vector from the 5′ end. The culture supernatants were purified on NI-NTA columns (Qiagen) to produce a band which was approximately ~19 kDa and was identified as a putative 6His-rgpTNF-*α* in the eluted fractions by SDS-PAGE analysis. Importantly, transfection of HEK-293 EBNA cells with the empty pCEP-Pu vector (not containing the gpTNF-*α* gene generated using Klenow polymerase) did not generate any protein band when analyzed on a Coomassie Blue stained SDS-gel (Figure 1(a)). The eukaryotic expressed rgpTNF-*α* was confirmed by its specific and strong binding to antiserum obtained from rabbits immunized with prokaryotic expressed rgpTNF-*α* protein (Figure 1(b)).

### 3.3. Analysis of Posttranslational Modifications in Eukaryotic-Expressed rgpTNF-*α*

The expression of eukaryotic genes cloned in* E. coli* lack posttranslational modifications. To determine whether such modifications were introduced into rgpTNF-*α* by a eukaryotic expression system, the eukaryotic-expressed rgpTNF-*α* protein was subjected to LC-MS/MS. Analysis by Proteome software showed that 83% of the mature peptide region was detected and there are no posttranslational modifications in those regions (see Table  1 in Supplementary Material available online at http://dx.doi.org/10.1155/2015/619480). However, intentional modifications such as carbamidomethylation and deamidation were seen (Supplementary Table  1). The carbamidomethylation is a result of reduction and alkylation. It is an intentional carboxymethylation on Cys to prevent reformation of Cys-Cys disulfides and allow complete denaturation and digestion. The deamidation is a common artifact of protein purification. Glutamine, and sometimes asparagines, are easily deamidated as is oxidation of methionine (proteins are oxidized when exposed to oxidizing atmosphere after cell disruption and the cell is a reducing environment).

### 3.4. Biological Activity of Prokaryotic- and Eukaryotic-Expressed rgpTNF-*α*

**Figure 2:**
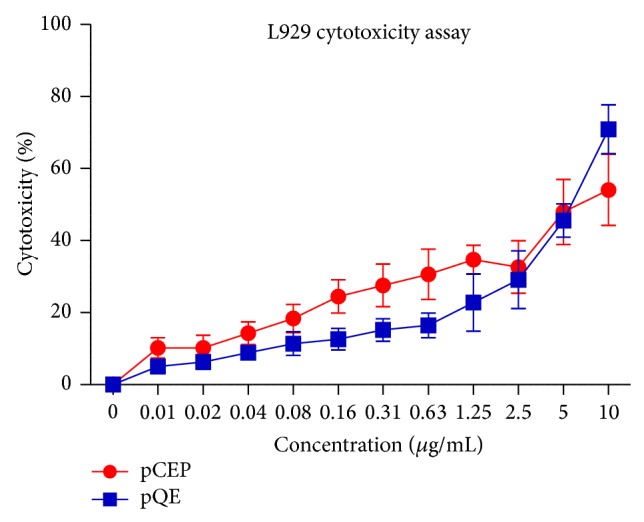
Bioactivity of prokaryotic and eukaryotic expressed guinea pig TNF-*α*. Prokaryotic and eukaryotic expressed rgpTNF-*α* proteins in the concentration ranging from 0 to 10 *μ*g/mL were analyzed for their cytotoxicity on L-929 fibroblasts and the percentage of cytotoxicity was calculated.

Both the prokaryotic- and eukaryotic-expressed rgpTNF-*α* exhibited cytotoxicity on L929 fibroblasts, a standard and widely used assay for TNF-*α* biological activity [28] (Figure 2). A dose-dependent increase in cytotoxicity was observed with both of the recombinant proteins (Figure 2). Importantly, there was no significant difference in dose-dependent cytotoxicity between prokaryotic and eukaryotic-expressed rgpTNF-*α*. Thus, prokaryotic-expressed rgpTNF-*α* is as biologically active as eukaryotic-expressed rgpTNF-*α* at the same concentration in this standard bioassay.

## 4. Discussion

This is the first comparative biochemical characterization of rgpTNF-*α* produced in prokaryotic and eukaryotic expression systems. TNF-*α* contributes to both host resistance and disease pathology in TB and other chronic diseases [1]. The importance of TNF-*α* in the control of latent or persistent mycobacteria has been revealed by the high risk of reactivation TB observed in patients undergoing anti-TNF-therapy for autoimmune diseases [29]. Since approximately one-third of the world's population is latently infected with TB [30] there is immense need to understand the role of this cytokine in the control of latent infection and precisely how its pharmacological suppression results in reactivation of TB. Such an understanding could drive the development of novel therapeutics which could be used to suppress the detrimental effects of TNF-*α* in autoimmune diseases without interfering with the essential host defense mechanisms that keep* M. tuberculosis* in check [31]. Such studies will benefit greatly from the availability of small animal models in which detailed mechanistic investigations of TNF-*α* at the molecular and cellular level can be conducted. The guinea pig is widely accepted to be the small animal model of choice for TB, however, knowledge of the basic biology of guinea pig cytokines and reagents with which to study them are only now developing [32].

In our work by us and others, the function of gpTNF-*α* was examined in a series of studies in which rgpTNF-*α* and polyclonal antisera were used to enhance or suppress, respectively, the cytokine's functions* ex vivo* in guinea pig phagocytic cells or* in vivo* in whole animals infected with virulent* M. tuberculosis* [7, 17, 18, 24]. The choice of a prokaryotic expression system for those early studies was based upon the advantages of ease of manipulation of bacteria* versus* eukaryotic cells, and the high yield of protein [20]. While those studies yielded important new information about the contributions of TNF-*α* to TB resistance, there was always a concern that the prokaryotic-expressed rgpTNF-*α* did not contain posttranslational modifications which might be important for TNF-*α* function in the guinea pig. Therefore, we undertook the task of expressing rgpTNF*α* in a eukaryotic expression system and examining the protein for such modifications.

**Figure 3:**
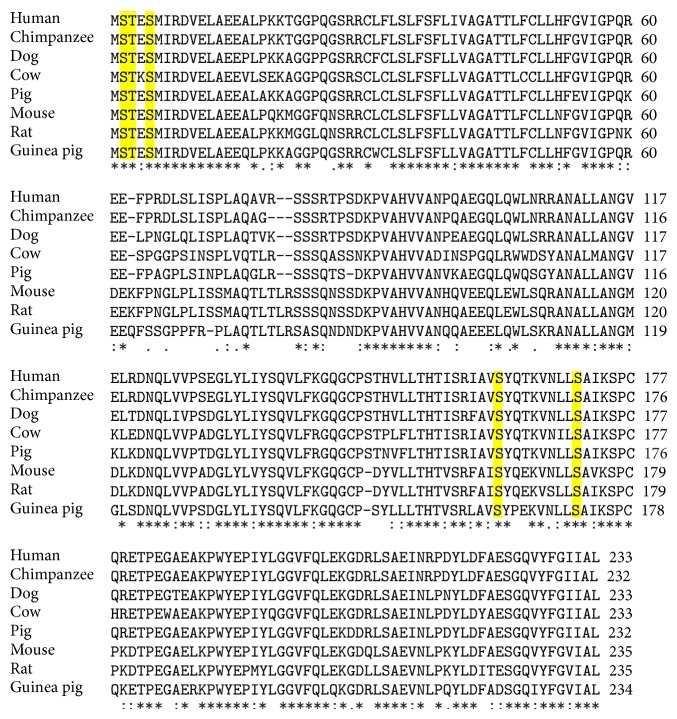
Amino acid sequence comparison of TNF-*α* from different species showing the presence of conserved putative phosphorylation sites. Amino acid sequences of human (AAA63207), chimpanzee (ABM91951), Squirrel monkey (AAK92045), Cow (AAA19011), Pig (AAA74410), Woodchuck (AY253723), Mouse (AAA39275), Rat (AAA41425), and Guinea pig (JN020146) were aligned by EBI Clustal W. Shading emphasizes identical amino acids and numbers on the right represent the position of amino acid.

Posttranslational modifications have significant effects on the structure and function of many mammalian proteins [33, 34]. No published information is available on the posttranslational modification of any guinea pig protein. Posttranslational modifications are reported to exert significant effect on the activity of cytokine proteins [35, 36]. In recombinant TNF-*α* from humans, mice and rats, three of the five potential posttranslational modification sites are in the signal peptide region, where as the other two are in the mature peptide region. (http://www.phosphosite.org/proteinAction.do?id=8542247&showAllSites=true). Interestingly, all the five potentially phosphorylated amino acids are also conserved in the guinea pig (second, third, and fifth amino acids -S, T, S) as well as other mammalian species (Figure 3). However, since the mature peptide region was cloned without the need for the signal peptide region, the first three modifications were not observed. Peptides corresponding to the last two modifications were obtained in our analysis but posttranslational modifications were not observed in our analysis.

We observed no posttranslational modifications in our eukaryotic-expressed rgpTNF-*α* and no difference in the dose-dependent biological function in the standard L929 cell cytotoxicity assay (Figure 2; Supplementary Table  1). In addition, rabbit polyclonal antisera developed in response to prokaryotic-expressed rgpTNF*α* cross-reacted strongly with the eukaryotic-expressed protein (Figure 1). Additional work with the recombinant proteins, including another functional assay to validate these findings, was not possible because the laboratory in which these studies were performed has closed due the retirement of the principal investigator. Thus, we conclude that the prokaryotic expressed rgpTNF-*α* can be used in future studies without concern that the expression system has failed to introduce modifications which alter the protein's biological function significantly. The prokaryotic expression system is preferred because of the ease of manipulation and the relatively higher yield. In studies comparing the yield of rgpTNF-*α* in the two expression systems, we observed at least 20-fold increase in the protein yield from* E. coli* compared to the HEK cells (Data not shown). Given the importance of the guinea pig as a small animal model of TB and other diseases [37, 38], we believe that our observations will facilitate future studies of the role of TNF-*α* using recombinant protein expressed in prokaryotic systems.

## Supplementary Material

Supplementary Table 1. Post-translational modification analysis of eukaryotic expressed rgpTNF-α by NanoLC-MS/MS.
