# Supplementary material for: Molecular and Biochemical Characterization of Recombinant Guinea Pig Tumor Necrosis Factor-Alpha
Source: Mediators Inflamm. 2015 Apr 27;2015:619480. doi: 10.1155/2015/619480 (PMC4427127; doi:10.1155/2015/619480)
Supplement: Supplementary file 1 — Supplementary Table 1. Post-translational modification analysis of eukaryotic expressed rgpTNF-α by NanoLC-MS/MS. [file 619480.f1.pdf]

Supplementary Table 1

Peptide sequence	Previous amino acid	Next amino acid	Mascot Identity score	Mascot Delta Ion score	Modifications identified by spectrum
ANGMGLSDNQL	L	V	63.5	6.2	Oxidation (+16)
ANGMGLSDNQLVVPSDGLY	L	L	0	0	
ANGMGLSDNQLVVPSDGLY	L	L	0	0	Oxidation (+16), Deamidation (+1)
ANGMGLSDNQLVVPSDGLY	L	L	0	0	Deamidation (+1)
ANGMGLSDNQLVVPSDGLY	L	L	0	0	
AVSYPEKVNL	L	L	61	7.5	
AVSYPEKVNLL	L	S	60.9	9.24	Deamidation (+1)
AVSYPEKVNLL	L	S	60	5.16	
DFADSGQIY	L	F	62.8	12.8	
DFADSGQIYFGVIAL	L	-	0	0	
DRLSAEVNLPQYLDFADSGQIY	G	F	0	0	
FKGQGCPSY	L	L	62.9	7.94	Carbamidomethyl (+57)
FKGQGCPSY	L	L	62.9	0	Carbamidomethyl (+57)
FKGQGCPSYL	L	L	0	0	Carbamidomethyl (+57)
FKGQGCPSYL	L	L	63.6	9.92	Carbamidomethyl (+57)
FQLQKGDRLSAEVNLPQYL	V	D	0	0	
GDRLSAEVNLPQYLDFADSGQIY	K	F	0	0	
GLSDNQLVVPSDGLY	M	L	0	0	
GLSDNQLVVPSDGLY	M	L	0	0	
GLSDNQLVVPSDGLY	M	L	0	0	
GLSDNQLVVPSDGLY	M	L	0	0	
GLSDNQLVVPSDGLY	M	L	0	0	
KGDRLSAEVNLPQYLDFADSGQIY	Q	F	0	0	

KGDRLSAEVNLPQYLDFADSGQIY	Q	F	0	0	
KGQGCPSY	F	L	61.4	-0.03	Carbamidomethyl (+57)
KGQGCPSY	F	L	61.6	7.02	Deamidation (+1), Carbamidomethyl (+57)
KGQGCPSY	F	L	61.4	13.8	Carbamidomethyl (+57)
KGQGCPSY	F	L	61.6	0	Deamidation (+1), Carbamidomethyl (+57)
KGQGCPSYL	F	L	62.5	3.59	Carbamidomethyl (+57)
LANGMGLSDNQL	L	V	63.9	20.8	Deamidation (+1), Oxidation (+16)
LANGMGLSDNQLVVPSDGLY	L	L	0	0	Deamidation (+1), Oxidation (+16), Deamidation (+1)
LANGMGLSDNQLVVPSDGLY	L	L	0	0	Deamidation (+1), Oxidation (+16)
LANGMGLSDNQLVVPSDGLY	L	L	0	0	Deamidation (+1), Deamidation (+1)
LANGMGLSDNQLVVPSDGLY	L	L	0	0	Deamidation (+1)
LANGMGLSDNQLVVPSDGLY	L	L	0	0	Deamidation (+1)
LANGMGLSDNQLVVPSDGLY	L	L	0	0	Deamidation (+1), Deamidation (+1)
LANGMGLSDNQLVVPSDGLY	L	L	0	0	Deamidation (+1)
LLTHTVSRL	L	A	53.7	9.3	
LLTHTVSRL	L	A	53.3	15	
LLTHTVSRLAVSYPEKVNLL	L	S	0	0	

LQKGDRLSAEVNLPQYL	Q	D	0	0	
LSAIKSPCQKETPEGAERKPW	L	Y	63	4.29	Carbamidomethyl (+57)
LSDNQLVVPSDGLY	G	L	0	0	
LTHTVSRL	L	A	55.5	5.87	
NQLVVPSDGLY	D	L	0	0	
QKETPEGAERKPW	C	Y	0	0	Pyro-cmC (-17)
QKGDRLSAEVNLPQYLDF	L	A	0	0	Pyro-cmC (-17)
QKGDRLSAEVNLPQYLDFADSGQIY	L	F	0	0	Pyro-cmC (-17)
QKGDRLSAEVNLPQYLDFADSGQIY	L	F	0	0	Deamidation (+1)
QKGDRLSAEVNLPQYLDFADSGQIY	L	F	0	0	Pyro-cmC (-17), Deamidation (+1)
QKGDRLSAEVNLPQYLDFADSGQIY	L	F	0	0	Pyro-cmC (-17)
QLQKGDRLSAEV	F	N	0	0	Pyro-cmC (-17)
QLQKGDRLSAEVNLPQY	F	L	0	0	Pyro-cmC (-17)
QLQKGDRLSAEVNLPQYL	F	D	0	0	Pyro-cmC (-17)
QLQKGDRLSAEVNLPQYL	F	D	0	0	Pyro-cmC (-17)
QLQKGDRLSAEVNLPQYL	F	D	0	0	Deamidation (+1)
QLQKGDRLSAEVNLPQYL	F	D	0	0	Pyro-cmC (-17)
QLQKGDRLSAEVNLPQYLDF	F	A	0	0	Deamidation (+1)
QLQKGDRLSAEVNLPQYLDF	F	A	0	0	Pyro-cmC (-17), Deamidation (+1)
QLQKGDRLSAEVNLPQYLDF	F	A	0	0	Pyro-cmC (-17), Deamidation (+1), Deamidation (+1)
QLQKGDRLSAEVNLPQYLDFAD	F	S	0	0	Pyro-cmC (-17)
QLQKGDRLSAEVNLPQYLDFADS	F	G	0	0	Pyro-cmC (-17)
QLQKGDRLSAEVNLPQYLDFADSG	F	Q	0	0	Pyro-cmC (-17)
QLQKGDRLSAEVNLPQYLDFADSGQIY	F	F	0	0	
QLQKGDRLSAEVNLPQYLDFADSGQIY	F	F	0	0	Pyro-cmC (-17)
QLQKGDRLSAEVNLPQYLDFADSGQIY	F	F	0	0	Pyro-cmC (-17)

QLQKGDRLSAEVNLPQYLDFADSGQIY	F	F	0	0	
QLQKGDRLSAEVNLPQYLDFADSGQIY	F	F	0	0	Pyro-cmC (-17)
QLQKGDRLSAEVNLPQYLDFADSGQIY	F	F	0	0	Pyro-cmC (-17)
QLQKGDRLSAEVNLPQYLDFADSGQIY	F	F	0	0	Pyro-cmC (-17)
RLAVSYPEKVNL	S	L	0	0	
RLAVSYPEKVNLL	S	S	0	0	
RLSAEVNLPQYLDFADSGQIY	D	F	0	0	
SAEVNLPQYL	L	D	62	6.55	
SAEVNLPQYLDF	L	A	64.6	0.86	Deamidation (+1)
SAEVNLPQYLDF	L	A	64.2	9.9	
SAEVNLPQYLDFADSGQIY	L	F	0	0	Deamidation (+1)
SAEVNLPQYLDFADSGQIY	L	F	0	0	Deamidation (+1)
SAEVNLPQYLDFADSGQIY	L	F	0	0	Deamidation (+1)
SAEVNLPQYLDFADSGQIYF	L	G	0	0	Deamidation (+1), Deamidation (+1)
SAIKSPCQKETPEGAERKPW	L	Y	65.7	4.35	Carbamidomethyl (+57), Deamidation (+1)
SAIKSPCQKETPEGAERKPW	L	Y	64.5	18.9	Carbamidomethyl (+57)
SAIKSPCQKETPEGAERKPW	L	Y	65	3.38	Carbamidomethyl (+57), Deamidation (+1)
SAIKSPCQKETPEGAERKPW	L	Y	64.9	14.6	Carbamidomethyl (+57), Deamidation (+1)
SAIKSPCQKETPEGAERKPW	L	Y	64.6	5.01	Carbamidomethyl (+57)
SAIKSPCQKETPEGAERKPW	L	Y	64.6	22.4	Carbamidomethyl (+57)
SAIKSPCQKETPEGAERKPW	L	Y	65	16.8	Carbamidomethyl (+57), Deamidation (+1)
SAIKSPCQKETPEGAERKPW	L	Y	65.1	13.9	Carbamidomethyl

					(+57), Deamidation (+1)
SAIKSPCQKETPEGAERKPW	L	Y	64.6	34.3	Carbamidomethyl (+57)
SAIKSPCQKETPEGAERKPW	L	Y	64.5	27	Carbamidomethyl (+57)
SAIKSPCQKETPEGAERKPW	L	Y	64.9	25	Carbamidomethyl (+57), Deamidation (+1)
SAIKSPCQKETPEGAERKPW	L	Y	65	35.4	Carbamidomethyl (+57), Deamidation (+1)
SDNQLVVPSDGLY	L	L	64.2	10.3	Deamidation (+1)
SDNQLVVPSDGLY	L	L	64	0.54	
SDNQLVVPSDGLY	L	L	64.1	25.5	
SDNQLVVPSDGLYL	L	I	0	0	
SKRANALLANGMGL	L	S	62.8	12.7	Deamidation (+1), Deamidation (+1), Oxidation (+16)
THTVSRLAVSYPEKVNLL	L	S	58.2	0	
TVSRLAVSYPEKVNLL	H	S	0	0	
VVANQQAEEELQW	H	L	0	0	
VVANQQAEEELQWL	H	S	0	0	
VVPSDGLY	L	L	60.9	0	
